# Sarcomere dynamic instability and stochastic heterogeneity drive robust cardiomyocyte contraction

**DOI:** 10.7554/eLife.97321

**Published:** 2026-09-14

**Authors:** Daniel Haertter, Lara Hauke, Til Driehorst, Kengo Nishi, Wolfram H Zimmermann, Christoph F Schmidt

**Affiliations:** 1 Institute of Pharmacology and Toxicology, University Medical Center Göttingen Göttingen Germany; 2 DZHK (German Center for Cardiovascular Research), partner site Lower Saxony Göttingen Germany; 3 CIDAS (Campus Institute Data Science), University of Göttingen Göttingen Germany; 4 Department of Physics and Soft Matter Center, Duke University Durham United States; 5 Third Institute of Physics, Faculty for Physics, University of Göttingen Göttingen Germany; 6 Cluster of Excellence "Multiscale Bioimaging: from Molecular Machines to Networks of Excitable Cells" (MBExC), University of Göttingen Göttingen Germany; 7 Fraunhofer Institute for Translational Medicine and Pharmacology Göttingen Germany; Institut Curie France; Max Planck Institute for Heart and Lung Research Germany

**Keywords:** cardiomyocytes, dynamic instability, stochastic heterogeneity, force–velocity relationship, sarcomere popping, tug-of-war, Human

## Abstract

Cardiac contraction is driven by the collective action of cardiomyocytes (CMs) that contain parallel bundles of myofibrils consisting of linear chains of sarcomeres, the basic force-generating units. The dynamics of individual sarcomeres within intact CMs remain incompletely understood. While most models assume uniform, synchronized contractions, recent studies hint at unexpected heterogeneity, whose origins and significance are not yet clear. By combining the culture of fluorescent sarcomere-reporter human induced pluripotent stem cell-derived CMs on micropatterned soft gels of different stiffness (5–85 kPa) with AI-based tracking of sarcomere motion, we found that increasingly stiff substrates inhibited overall CM contraction, but, surprisingly, did not diminish individual sarcomere dynamics. Instead, sarcomeres competed in a tug-of-war, causing increasing heterogeneity, including rapid length oscillations and overextensions (popping). Statistical analysis showed that the heterogeneous dynamics were not caused by static structural differences but were largely stochastic. Stochastic heterogeneity is thus an intrinsic property of cardiac sarcomeres and likely mediates the adaptation of CM contractility to mechanical constraints. A mesoscopic model of coupled sarcomeres shows that these phenomena can be explained by a non-monotonic force–velocity relationship and stochastic fluctuations, where dynamic instability at a critical yielding force creates heterogeneity. Stochastic heterogeneity compensates for structural disorder by randomizing yield events beat-to-beat, preventing damage to specific sarcomeres. Our findings recast cardiac sarcomeres as active, dynamically unstable, and stochastic units engaged in a stochastic tug-of-war, where transient, velocity-dependent forces dominate. We propose that pathological disorder in cardiomyopathy drives a transition from protective stochastic fluctuations to more deterministic, persistently overloaded sarcomeres.

## Introduction

**Figure 1.**
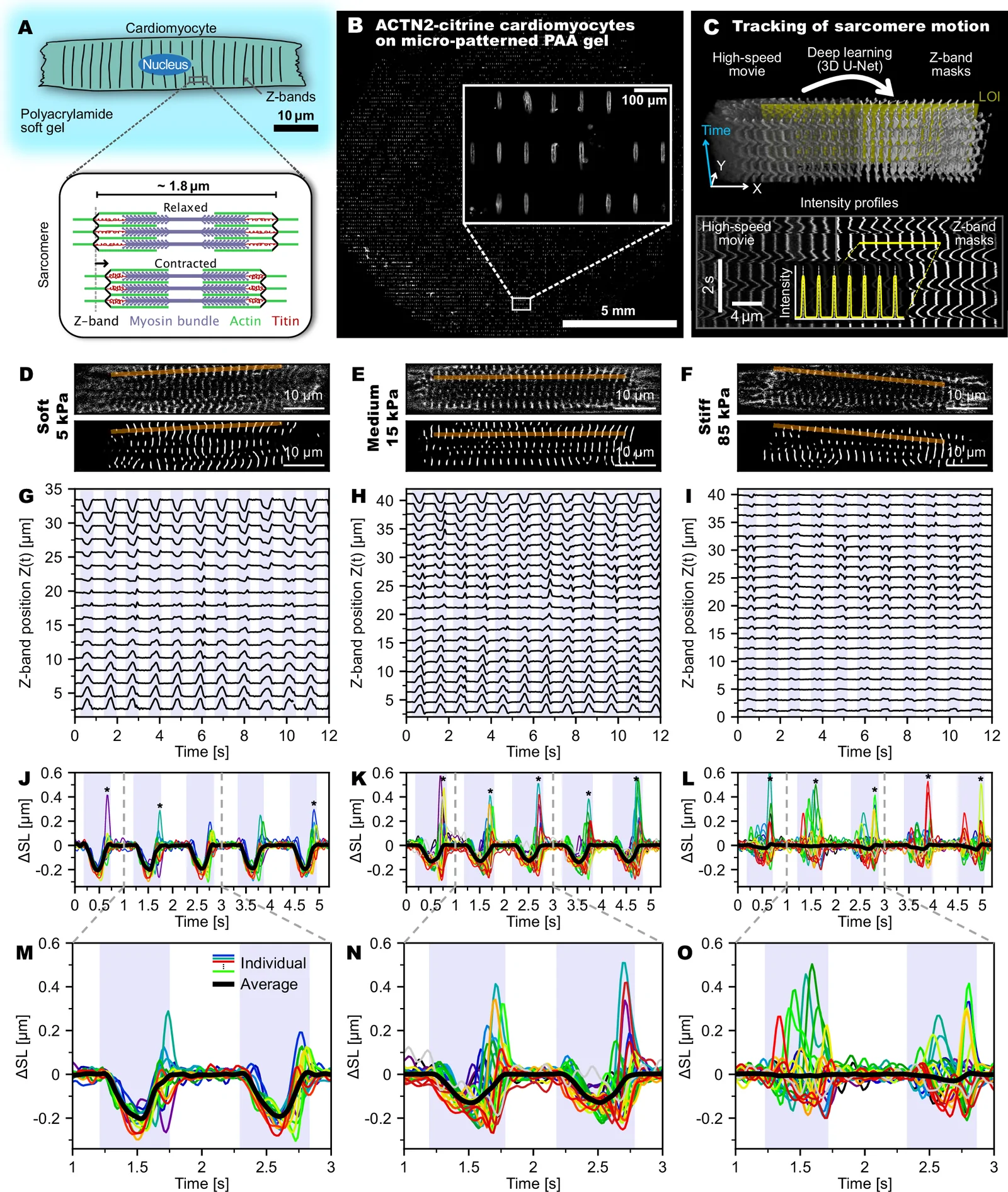
Sarcomere tracking in genetically engineered Z-line labeled induced pluripotent stem cell (iPSC)-derived cardiomyocytes (CMs) on micropatterned soft gels. (**A**) Sketch of a human CM adhering to a micropatterned gel (top) and sarcomere structure in relaxed and contracted state (bottom). (**B**) ACTN2-Citrine CMs (culture day 20) on a polyacrylamide gel substrate (Young’s modulus: 15 kPa), patterned with rectangular stripes of Synthemax (70 × 10 µm). More than 50% of the stripes were typically occupied by single CMs. Inset: zoomed-in view. (**C**) Tracking of sarcomere motion: (Top) Blend of high-speed confocal movie of a CM adherent to a 15-kPa substrate (left) and deep-learning (3D U-Net)-based segmentation of sarcomere Z-bands (right) with line of interest (LOI, yellow). (Bottom) Intensity extracted along LOI. Inset shows representative section of the Z-band intensity profile. (**D, E**) Confocal images of representative ACTN2-Citrine-labeled CMs with corresponding Z-band segmentation and LOIs (red lines). (**G–I**) Z-band trajectories tracked along the LOI. (**J–O**) Overlay plots of single-sarcomere length change ∆*SL*(*t*) for all tracked sarcomeres in the marked LOIs (first 5 s in **J–L**, zoomed in **M–O**). Colored lines are individual sarcomere length changes; black lines display average length changes. Contraction intervals are marked by a blue background. Sarcomere popping events are marked with asterisks. Conditions (substrate stiffness): 5 kPa (**D, G, J, M**); 15 kPa (physiological) (**E, H, K, N**); 85 kPa (**F, I, L, O**).

Sarcomeres are the basic contractile cytoskeletal units of striated muscle, including cardiac muscle. In most species, sarcomeres are ~2 µm in length and consist of highly ordered bipolar bundles of myosin motor proteins interdigitated with actin filaments, tied together in the Z-bands (Figure 1A). Force generation by myosin interacting with actin is regulated by intracellular Ca^2+^ and fueled by chemical energy (ATP) (Maack et al., 2019). Sarcomeres are serially connected into myofibrils that span the length of the cell. A cardiomyocyte (CM) contains tens of parallel myofibrils. Overall CM contractility is a mesoscopic process that emerges from a very large number of coupled, non-linear, stochastic, elementary force generators. Despite extensive knowledge of single-molecule motor kinetics (Spudich, 2001; Huxley and Simmons, 1971) and whole-muscle mechanics (Gordon et al., 1966; Rassier, 2017), this intermediate mesoscopic scale—where tens of sarcomeres interact within a myofibril—remains largely unexplored in cardiac muscle. Sarcomeres are mechanically coupled in series, yet each is also an active, excitable unit undergoing rapid, transient activation–relaxation cycles. Do these coupled sarcomeres maintain synchronized motion, or do stochastic fluctuations and non-equilibrium forces drive heterogeneous behavior?

**Figure 1—figure supplement 1.**
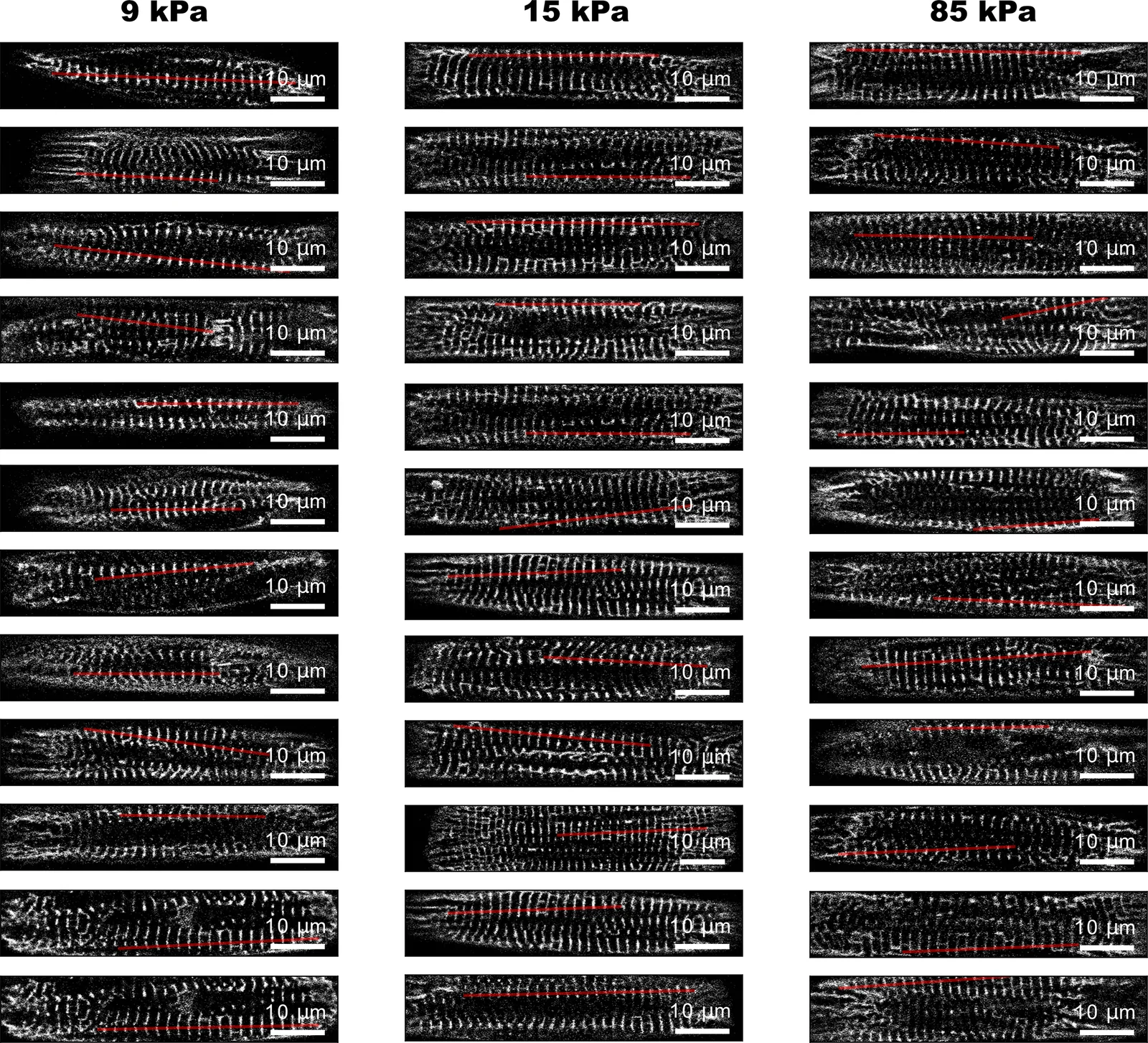
Representative cardiomyocyte (CM) selection with automatically and individually detected ROIs. CMs are randomly selected from the data sets for three substrate stiffnesses (9, 15, and 85 kPa); automatically determined ROIs depicted as red lines.

**Figure 1—figure supplement 2.**
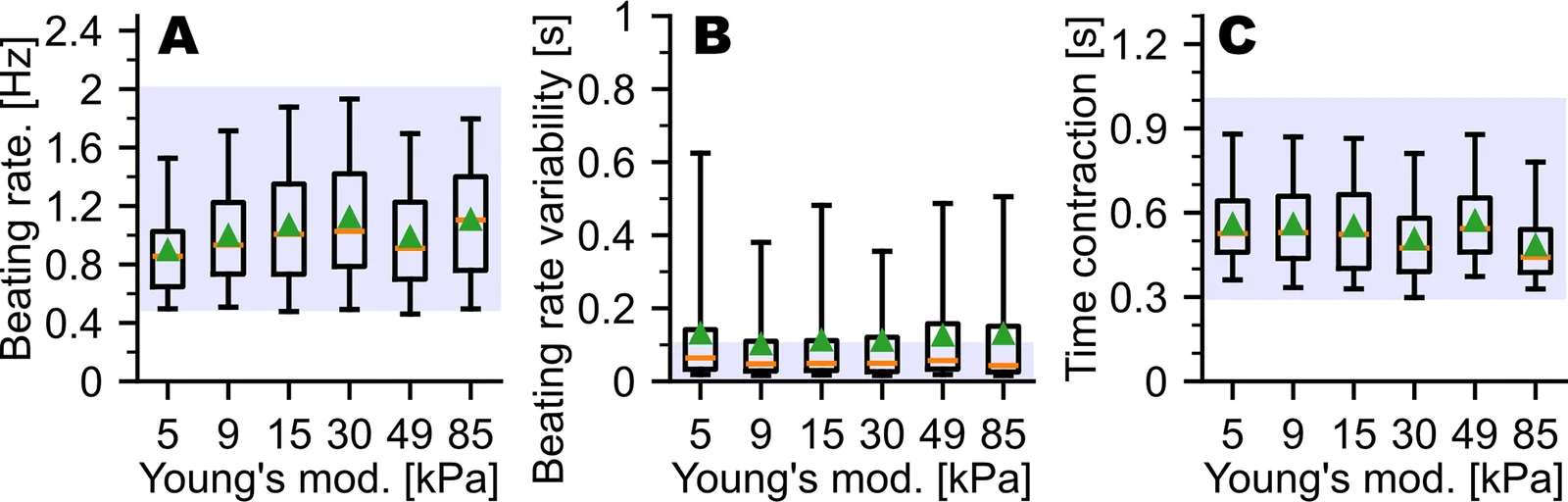
Effects of substrate elasticity on spontaneous beating of cardiomyocytes. (**A**) Spontaneous beating frequencies. (**B**) Beating irregularity: relative standard deviation of beating periods. (**C**) Average length of contraction cycles *T_c_*. (**A–C**) include data from 5085 lines of interest (LOIs). Boxes show quartiles, red lines the median, green triangles the mean, and whiskers the 5th and 95th percentiles of the distributions. Blue shaded intervals show included data.

Recent studies, utilizing in vivo imaging and advanced in vitro models, have begun to reveal notable heterogeneity and beat-to-beat variability at the sarcomere level—even as the average myofibril motion remains smooth and regular (Kobirumaki-Shimozawa et al., 2021; Li et al., 2023; Kobirumaki-Shimozawa et al., 2024). However, these observations have been limited to murine systems (≈5–8 Hz beating rate vs. ~1 Hz in humans). While sarcomere non-uniformity has a long history in skeletal muscle research (Edman, 1980; Telley et al., 2003; Moo et al., 2017; Givli, 2010), prevailing models of cardiac contraction have historically assumed that sarcomeres behave as uniform, synchronized units, with force generation determined primarily by the steady-state force–length relationship—an idea rooted in the Frank–Starling mechanism and length-dependent activation (Gordon et al., 1966; de Tombe et al., 2010).

Existing attempts to explain cardiac sarcomere heterogeneity remain largely qualitative, invoking static structural differences between sarcomeres—for example, in strength or resting length—especially pronounced in cardiomyopathy (Kobirumaki-Shimozawa et al., 2021; Li et al., 2023; Kobirumaki-Shimozawa et al., 2024; Månsson, 2014). Such models propose deterministic mechanisms: ‘weak’ sarcomeres are consistently stretched by ‘strong’ neighbors (Månsson, 2014), diastolic length variations dictate subsequent systolic interactions and potential role-switching between sarcomeres (Kobirumaki-Shimozawa et al., 2021; Kobirumaki-Shimozawa et al., 2024), or inherent length variability enables recruitment of more contracting sarcomeres upon cell lengthening through length-dependent activation (Li et al., 2023).

Crucially, these deterministic, primarily length-centric views neglect velocity-dependent active force generation, friction effects, and non-linearities. By assuming a continuous balance between passive and active forces dictated by steady-state relationships, they ignore the non-equilibrium nature of the beating heart, where adaptation kinetics and viscous drag are critical. Consequently, unraveling these dynamics requires novel approaches that resolve the actual motion of individual sarcomeres within their native physiological setting—inside intact, beating CMs—tightly integrated with quantitative modeling.

In this study, we cultured human fluorescent sarcomere-reporter human induced pluripotent stem cell (hiPSC)-derived cardiomyocytes (ACTN2-Citrine-CMs) on micropatterned gel substrates of different stiffnesses to investigate sarcomere dynamics under different mechanical boundary conditions. High-speed imaging, combined with AI-based analysis using our SarcAsM algorithm (Härtter et al., 2025), enabled us to track average and individual sarcomere motions within myofibrils with nanometer resolution.

Our analysis found strikingly complex dynamic behaviors, including rapid length oscillations, transient ‘popping’ events, and heterogeneous contraction patterns that varied both temporally and spatially along one myofibril. We show that this stochastic heterogeneity appears to arise from competitive, tug-of-war-like interactions among mechanically coupled sarcomeres within the myofibrils. To rationalize these observations, we developed a data-driven mesoscopic model which demonstrates that the emergent heterogeneity at the sarcomere level can be explained by stochastic fluctuations in conjunction with a non-monotonic force–velocity relation with a critical force threshold. These insights into stochasticity and dynamic instability at the sarcomere level challenge traditional paradigms of myocardial contraction.

## Results

### Individual ACTN2-Citrine hiPSC-derived CMs on micropatterned elastic substrates

To resolve sarcomere dynamics with high spatial and temporal resolution in PSC-derived CMs, we used a CRISPR-engineered induced pluripotent stem cell (iPSC) line, expressing a yellow fluorescing protein as ACTN2-Citrine N-terminal fusion protein after CM differentiation (Härtter et al., 2025). Seeding of differentiated CMs on soft elastic polyacrylamide gels functionalized with a micro-printed pattern (70 × 10 µm; Figure 1B) promoted anisotropic myofibril assembly in uniformly elongated CMs. Culturing cells on gels with defined elasticities (Young’s moduli: 5, 9, 15, 29, 49, and 85 kPa; Supplementary file 1A) allowed us to impose auxotonic loads on the cells on a scale considered to be relevant in vivo under physiological (10–20 kPa) and pathological (e.g., fibrosis; ≥30 kPa) conditions (Härtter et al., 2025). After a maturation period of 20–35 days, we recorded 20–30 s long movies of, in total, 1362 spontaneously beating CMs at a frame rate of 66 Hz (Video 1, Figure 1—figure supplement 1).

**Video 1.** Representative cardiomyocytes showing the effect of substrate stiffness on sarcomere heterogeneity. Real-time confocal movies of three representative ACTN2-Citrine labeled cardiomyocytes (16 ms frame time) on 5, 15, and 85 kPa substrates. Red lines show lines of interest (LOIs) for sarcomere motion tracking and analyses. Middle: Individual sarcomere length changes $\Delta SL_{i}\left (t\right)$ (colored lines) and average sarcomere length change $\overline{\Delta SL}\left (t\right)$ (black line) of myofibril ROIs shown above. Contraction intervals are shown in light blue. Black vertical line indicates time progress synchronized with movies above. Bottom: Length-change velocity phase portraits showing individual sarcomere trajectories (red traces), the average trajectory (black trace), and individual sarcomere positions (colored dots).

### Automated tracking of sarcomere motion at high spatial and temporal resolution

We used SarcAsM, an AI-based software tool for automated segmentation and analysis of sarcomere structure and dynamics (Härtter et al., 2025) for data evaluation. SarcAsM automatically identified 5085 lines of interest (LOIs), up to 4 per cell, with well-organized registered sarcomeres (≥10 sarcomeres; Figure 1C, Figure 1—figure supplement 1). Along each LOI (~800 nm in width), an intensity kymograph of Z-band motion was extracted from deep-learning (3D U-Net) processed movies, allowing for robust and time-consistent segmentation of Z-bands in noisy high-speed movies (Figure 1C). This approach provided more accurate and robust localization and tracking of Z-band trajectories $Z_{i}\left (t\right)$ of individual sarcomeres than using raw microscopy data (~17 nm and 15 ms resolution, details see SarcAsM Härtter et al., 2025). From $Z_{i}\left (t\right)$, we obtained sarcomere length $SL_{i}\left (t\right)$, sarcomere length change $\Delta SL_{i}\left (t\right)$, and sarcomere velocity $V_{i}\left (t\right)$ of each sarcomere i as well as multi-sarcomere averages $\overline{SL}\left (t\right)$, $\overline{\Delta SL}\left (t\right)$, and $\overline{V}\left (t\right)$ for each LOI (details see SarcAsM Härtter et al., 2025; Figure 1D–F).

### CM and individual sarcomere contractility as a function of substrate stiffness

The hiPSC-CMs were beating spontaneously at a frequency of 0.91 ± 0.38 Hz, which was largely independent of the substrate stiffness (Figure 1—figure supplement 2A). Beat-to-beat intervals, though, showed increasing irregularities with increasing substrate stiffness (Figure 1—figure supplement 2B). Contraction durations $T_{c}$ shortened with increasing substrate stiffness (Figure 1—figure supplement 2C).

**Figure 2.**
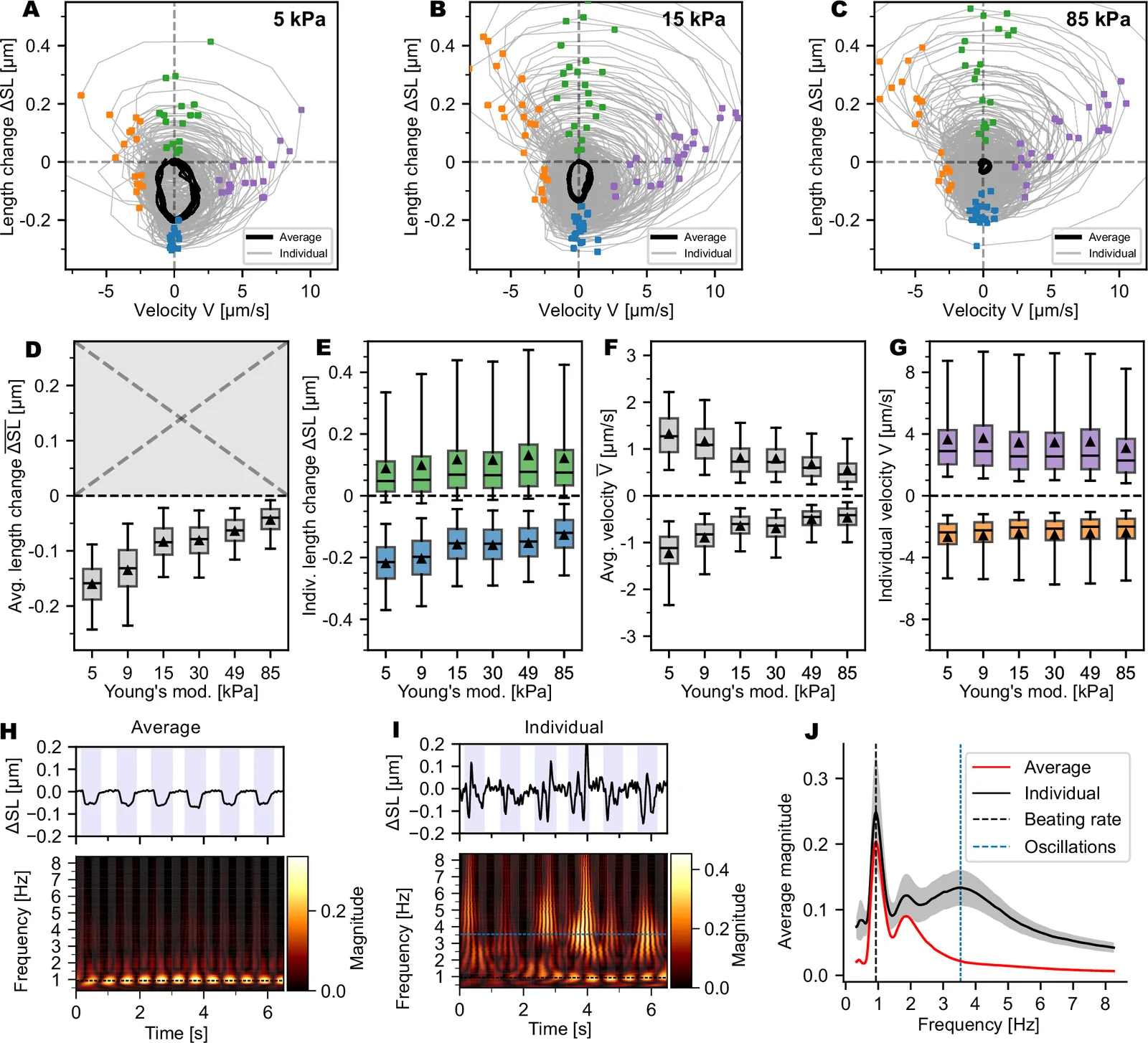
Analysis of sarcomere length change dynamics. (**A–C**) Phase-space plots of sarcomere length change ∆*SL* versus velocity *V* for three representative lines of interest (LOIs) from cardiomyocytes (CMs) on substrates of increasing stiffness (same LOIs as in Figure 1). Gray lines show individual sarcomere dynamics, black lines average dynamics. Colored dots mark maximal and minimal values of ∆*SL* and *V* for individual sarcomere trajectories, with colors corresponding to data in **E **and **G**. (**D**) Box plot of maximal average contractions $\overline{\Delta SL}_{-}$ as a function of substrate stiffness. Maximal average extensions $\overline{\Delta SL}_{+}$ are always close to 0 and not shown. (**E**) Box plot of maximal shortening and lengthening amplitudes $\Delta SL_{+\mathrm{/}-}$ of individual sarcomeres quantified in each contraction cycle. (**F**) Box plot of maximal average sarcomere lengthening and shortening velocities $\overline{V}_{+\mathrm{/}-}$. (**G**) Box plot of maximal individual sarcomere lengthening and shortening velocities $V_{+\mathrm{/}-}$. Boxes show quartiles, lines the median, triangles the mean, and whiskers the 5th and 95th percentiles of the distribution per condition. Each data point corresponds to the extremal value within one contraction cycle. To weigh each LOI equally, only the first 10 contraction cycles in each recording were considered. (**D–G**) combine data of 2321 LOIs from 1362 cells (5 kPa: 184, 9 kPa: 580, 15 kPa: 392, 29 kPa: 319, 49 kPa: 503, 85 kPa: 343). Statistical analysis was performed using the Kruskal–Wallis and Dunn’s post hoc tests, with significance set at p < 0.05. All differences were significant. (**H, I**) Timeseries and Morlet wavelet scalogram of average and single-sarcomere length changes $\Delta SL\left (t\right)$. The top plot displays average (**H**) and representative single (**I**) sarcomere length changes over time, with blue background marking contraction periods. The bottom plot presents the wavelet scalograms, depicting the evolution of frequency content in the signal over time, with the blue dashed line signifying the cell’s beating rate. (**J**) Comparison of time-averaged oscillation frequencies of average (red) and single (black) sarcomere length changes of one representative LOI, showing high-frequency intrinsic oscillatory motion of individual sarcomeres with frequencies of 3–4 Hz, which cancel out on the myofibril scale. For time-averaging of the frequency spectra, only the contraction intervals were included. The black curve shows mean ± SD of 16 sarcomeres in one representative LOI; the black dashed line shows the beating rate and the blue dashed line the peak of the oscillation frequency distribution.

In line with previous reports (Ribeiro et al., 2015; Engler et al., 2008; Hazeltine et al., 2012), total cell contraction amplitudes, quantified by the inward motion of the outermost Z-bands in myofibrils, substantially decreased with increasing substrate stiffness (Figure 1D–F). A surprising finding was that the length changes of individual sarcomeres $\Delta SL_{i}$ fell out of synchronization and became distinctly heterogeneous, deviating strongly from the average length change $\overline{\Delta SL}$ with increasing substrate stiffness. Sarcomeres also showed more singular large-amplitude extensions (sarcomere popping) far beyond their resting lengths, often very pronounced at the end of contractions (marked with asterisks in the bottom row of Figure 1D–F and large excursions in phase-space plots in Figure 2A–C). Despite the rapid and heterogeneous motion of individual sarcomeres, the emergent contraction at the myofibril scale remained regular and smooth on all substrates.

To analyze the deviations of individual sarcomere dynamics from the myofibril average during contraction cycles, we collected minima and maxima of ∆*SL*, that is, maximal contraction and elongation amplitudes, and minima and maxima of *V* for each contraction cycle for both individual sarcomeres and averages over all sarcomeres in a given LOI (Figure 2A–G). In total, we analyzed a data set of 5085 LOIs recorded from 1362 cells, each over at least 10 contraction cycles. We excluded irregularly beating (beat-to-beat variability >0.1 s) and very rapidly (>2 Hz) or slowly (<0.5 Hz) beating cells. We further limited the analysis to LOIs spanning whole myofibrils, where the average length change was strictly negative, that is, showing contraction. Out of the full data set of 5085 LOIs, 2321 LOIs met these criteria. The majority of eliminated LOIs were from irregularly beating cells (Figure 1—figure supplement 2B). This selection ensured that we focused on rhythmically and uniformly contracting cells. As expected, maximal average contraction amplitudes $\overline{\Delta SL}_{-}$, measured at the peaks of shortening in each contraction cycle, were the largest (0.16 ± 0.05 µm) on the softest (5 kPa) substrates and were close to zero (0.04 ± 0.03 µm) on the hardest substrates (85 kPa; Figure 2D). Maximal contraction amplitudes $\Delta SL_{-}$ of individual sarcomeres in each contraction cycle were also largest on 5 kPa substrates at 0.22 ± 0.09 µm and declined to 0.13 ± 0.07 µm on 85 kPa (Figure 2E). Note that the decline of maximal individual sarcomere shortening ($\Delta SL_{-}$) by ~30% from 5 to 85 kPa was far less than the ~75% decline of the average sarcomere contraction ($\overline{\Delta SL}_{-}$). In addition, while myofibrils never elongated beyond resting length, as expected for auxotonic contractions, individual sarcomeres frequently elongated well beyond their resting lengths during contraction cycles (median $\Delta SL_{+}$ = 0.05 µm). The distributions of maximal sarcomere extension amplitudes ($\Delta SL_{+}$) were remarkably similar between substrate conditions (Figure 2E).

Next, we evaluated the effect of substrate elasticity on average and individual sarcomere contraction and extension velocities. The average extremal velocities $\overline{V}_{+}$ and $\overline{V}_{-}$ followed the same trend as the extremal average length changes $\overline{\Delta SL}_{+}$ and $\overline{\Delta SL}_{-}$ and were the largest on 5 kPa and declined steadily and strongly with increasing substrate stiffness (from 5 to 85 kPa by ~60% for both contraction and elongation; Figure 2F). The single-sarcomere extremal contraction and extension velocities $V_{-}$ and $V_{+}$, in contrast, were far less affected by substrate elasticity and differed only by at most ~10% ($V_{-}$) and ~20% ($V_{+}$) for the various substrate conditions (Figure 2G). Note that the maximal extension velocities were 29–38% larger than the maximal absolute contraction velocities, showing a strong asymmetry between single-sarcomere contraction and extension dynamics.

### High-frequency oscillatory motion of individual sarcomeres

Unlike the average sarcomere motion with clearly distinguishable contraction and relaxation phases, single sarcomeres exhibited rapid switching between slow contractile and fast extensile motions with sometimes multiple shortening and lengthening phases within one contraction cycle (Figures 1D-F and 2H, I). To quantify this phenomenon, we analyzed the oscillation frequencies of the average and individual length changes $\Delta SL\left (t\right){\ }$ over time, utilizing Morlet wavelet analysis, a method that makes it possible to extract the instantaneous oscillation frequencies of a signal. For average sarcomere length changes $\overline{\Delta SL}\left (t\right),$ the analysis revealed a narrow range of frequencies surrounding the beating rate in the scalogram (Figure 2H, I). For individual sarcomeres, in contrast, we found a broader range of frequencies, with significant peaks occurring at the dominant and macroscopically visible whole-cell beating rate as well as a large secondary peak at 3 ± 0.5 Hz (Figure 2J). This distinctive high-frequency oscillation was consistently observed across numerous LOIs and appeared to be independent of the beating rate. The intrinsic oscillatory motion of individual sarcomeres suggests a mechanism of active actomyosin powered contraction coupled to rapid lengthening in a relaxation oscillator-like behavior (Strogatz, 2018).

### Correlation analysis identifies stochastic and static heterogeneity among sarcomeres

**Figure 3.**
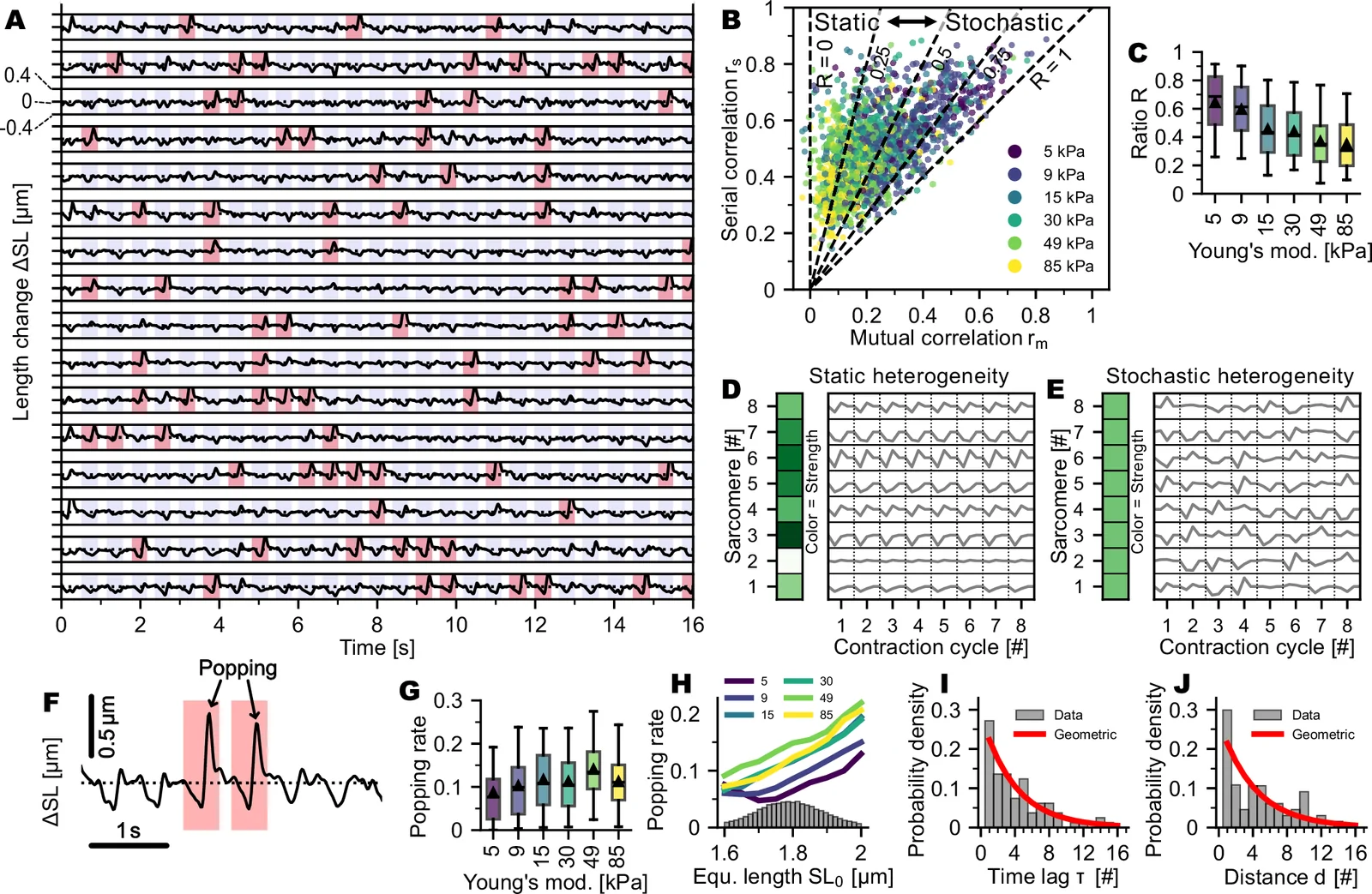
Static versus stochastic heterogeneity in the popping dynamics. (**A**) Representative timeseries of sarcomere length changes Δ*SL* of a myofibril in one representative cardiomyocyte on a 30-kPa substrate. Popping events, defined as sarcomere elongations beyond 0.25 μm within one contraction cycle, are marked in red. Contraction intervals are marked with a blue background. (**B**) Correlation analysis showing mutual correlation ($r_{m}$) versus serial correlation ($r_{s}$). The *x*-axis shows the mutual correlation of motion between different sarcomeres (*i* ≠ *j*), the *y*-axis shows the serial correlation of different cycles of one sarcomere (*i* = *j*, *k* ≠ *l*). Dashed lines delineate regions of static heterogeneity (left) and stochastic heterogeneity (right). Data points are colored by substrate stiffness (5–85 kPa). (**C**) Ratio R between average mutual and serial correlation of Δ*SL*, serving as a measure for the degree of stochasticity in motions, for different substrate stiffnesses. Illustrative computer-generated sketches of sarcomere length changes Δ*SL* in different contraction cycles for purely static heterogeneity (**D**) and purely stochastic heterogeneity (**E**). Color bar denotes sarcomere contractile strength. (**F**) Zoomed view of consecutive experimentally observed contraction cycles showing popping events (red shaded regions) where sarcomeres elongated beyond the threshold of 0.25 µm during contraction. (**G**) Overall popping frequencies (in units of 1/contraction cycle) for different substrate conditions. (**H**) Popping frequency as a function of sarcomere equilibrium length *SL*₀. Lines show averages for different substrate stiffnesses, with underlying distribution of equilibrium lengths shown below in gray. Probability density distributions of time lag (**I**) and distance (**J**) between popping events for single line of interest (LOI) compared with corresponding geometric distributions (red lines). **Data and statistics:** Panels B, C, G–J show data from 2321 LOIs (5 kPa: 184, 9 kPa: 580, 15 kPa: 392, 29 kPa: 319, 49 kPa: 503, 85 kPa: 343). Box plots show quartiles, mean (triangle) and median (line), with whiskers representing the 5th and 95th percentiles. Statistical analysis was performed using Kruskal–Wallis and Dunn’s post hoc tests, with significance set at p < 0.05. All differences were significant.

The observed heterogeneity of sarcomere dynamics, particularly on rigid substrates (Figure 3A), might be predetermined by static non-uniformities among sarcomeres (e.g., by differences in functional myosin numbers). To distinguish this possibility from stochastic heterogeneity, we extracted the time-resolved motion $\Delta SL_{i,k}\left (t\right)$ of each sarcomere *i* during each contraction cycle *k*. We then quantified the similarity between these motion patterns using the Pearson correlation coefficient. For two motion patterns $\Delta SL_{i,k}\left (t\right)$ and $\Delta SL_{j,l}\left (t\right)$, this coefficient is defined as

(1) $$\displaystyle r(i,j,k,l)= \frac{  \sum_{t=1}^{T} \left(\Delta SL_{(i,k)}(t)-\overline{\Delta SL}_{i,k}\right) \left(\Delta SL_{(j,l)}(t)-\overline{\Delta SL}_{j,l}\right) }{  \sqrt{\sum_{t=1}^{T} \left(\Delta SL_{(i,k)}(t)-\overline{\Delta SL}_{i,k}\right)^2} \sqrt{\sum_{t=1}^{T} \left(\Delta SL_{(j,l)}(t)-\overline{\Delta SL}_{j,l}\right)^2} },$$

where *t* represents the discrete time index (frame number) within a contraction cycle, *T* is the total number of frames capturing one contraction cycle, and $\overline{\Delta SL}$ denotes the mean value of the sarcomere length change over the cycle.

For each LOI, we calculated two correlation measures. The mutual correlation coefficient $r_{m}$ measures the synchrony between sarcomeres within the same contraction cycle, calculated as $r_{m}=\left \langle r\left (i,j,k,k\right)\right \rangle _{i\neq j}$, where the averaging is performed over all pairs of different sarcomeres (i≠j) within the same contraction cycle k. The serial correlation coefficient $r_{s}$ quantifies the consistency of individual sarcomeres across different contraction cycles, calculated as $r_{s}=\left \langle r\left (i,i,k,l\right)\right \rangle _{k\neq l}$, where the averaging is performed over all pairs of different contraction cycles (k≠l) for the same sarcomere i.

The serial and mutual correlations both were maximal on 5 kPa substrates and declined steadily with increasing substrate stiffness. The decrease of mutual correlation reflects the decrease of synchrony between sarcomeres due to the tug-of-war competition imposed by the rigid constraints on the myofibril level (Figure 3B). The decrease of serial correlation indicates an increased beat-to-beat variability of the motions of individual sarcomeres (Figure 3B). Interestingly, the mutual correlation declined more strongly than the serial correlation, by 50% to more than 90% from 5 to 85 kPa.

We introduce the ratio $R=r_{m}/r_{s}$ of serial to mutual correlation coefficients to distinguish static from stochastic heterogeneity (Figure 3B, C). When *R* = 1, the heterogeneity is completely stochastic. Random shuffling of sarcomeres (*i*, *j*) and contraction cycles (*k*, *l*) would in that case not affect *R*. When R≪1, the heterogeneity is largely static. In this case, mutual correlations are much smaller than serial correlations, indicating that the motion varies much more between sarcomeres than for a given sarcomere from one contraction cycle to another (Figure 3D, E). The broad distribution of *R* values for all examined LOIs shows that the heterogeneity was neither fully stochastic nor fully static but rather distributed across the full spectrum from stochastic to static (Figure 3C). We examined *R* for different substrate conditions and found a strong dependence on substrate elasticity for both length change and velocity correlations (Figure 3B). Heterogeneity was mostly stochastic for 5 and 9 kPa substrates and increasingly static for stiffer substrates with non-physiological elasticities.

### Sarcomere popping events are stochastically independent and not only occur in structurally ‘weak’ sarcomeres

Beyond the heterogeneous contraction patterns, we observed discrete mechanical instabilities in the form of sudden sarcomere elongations, mostly at the end of contractions, which we termed ‘popping’ events. Defined as sarcomere elongations exceeding 0.25 μm (Figure 3A, F), these events likely represent passive yielding where threshold tension triggers an avalanche-like release of myosin heads while neighboring sarcomeres continue to contract or relax in a more direct manner without overshoot.

The rate of popping events showed a clear dependence on substrate stiffness (Figure 3G). Stiffer substrates (49–85 kPa) led to higher popping rates than softer substrates (5–15 kPa), with median frequencies increasing from approximately 0.08 events per sarcomere per cycle on soft substrates to over 0.14 on the stiffest substrates. Importantly, even on the softest substrates, popping frequencies remained finite, indicating that sarcomere popping is an intrinsic feature rather than a pathological response to specific mechanical conditions. This baseline level of popping across all substrate stiffnesses demonstrates that mechanical instabilities are fundamental to sarcomere dynamics.

Popping frequency also correlated with sarcomere equilibrium length (*SL*), with longer sarcomeres exhibiting higher popping rates across all substrate conditions (Figure 3H). However, the distribution of equilibrium lengths (gray histogram in Figure 3H) was relatively tightly centered around 1.8 μm. Crucially, the popping rate remained consistently non-zero also across the entire range of equilibrium lengths, even in LOIs where length variability was minimal. This demonstrates that while resting length heterogeneity contributes to popping behavior, it is not the primary cause of these mechanical instabilities.

To assess the stochastic nature of popping events, we analyzed the distributions of time intervals and spatial distances between consecutive popping events. We assessed the distributions of distance (*d*) and time gaps (τ) for all LOIs and compared them with geometric distributions G(*k*) with respective event probabilities (Figure 3I, J). Using the Kolmogorov–Smirnov test, we could not reject the hypothesis that popping events are stochastically independent for 49% of LOIs with respect to distance and 47% of LOIs with respect to time gaps (p-value = 0.01). This substantial proportion of LOIs showing stochastically independent popping events demonstrates that popping has a stochastic component, independent of deterministic factors such as sarcomere strength heterogeneity or structural variations. While the remaining LOIs show non-random clustering patterns—likely reflecting the influence of local mechanical coupling or morphological heterogeneities—the underlying stochastic mechanism appears to be intrinsic to sarcomere mechanics itself.

### A mesoscopic Langevin framework for coupled sarcomere dynamics

**Figure 4.**
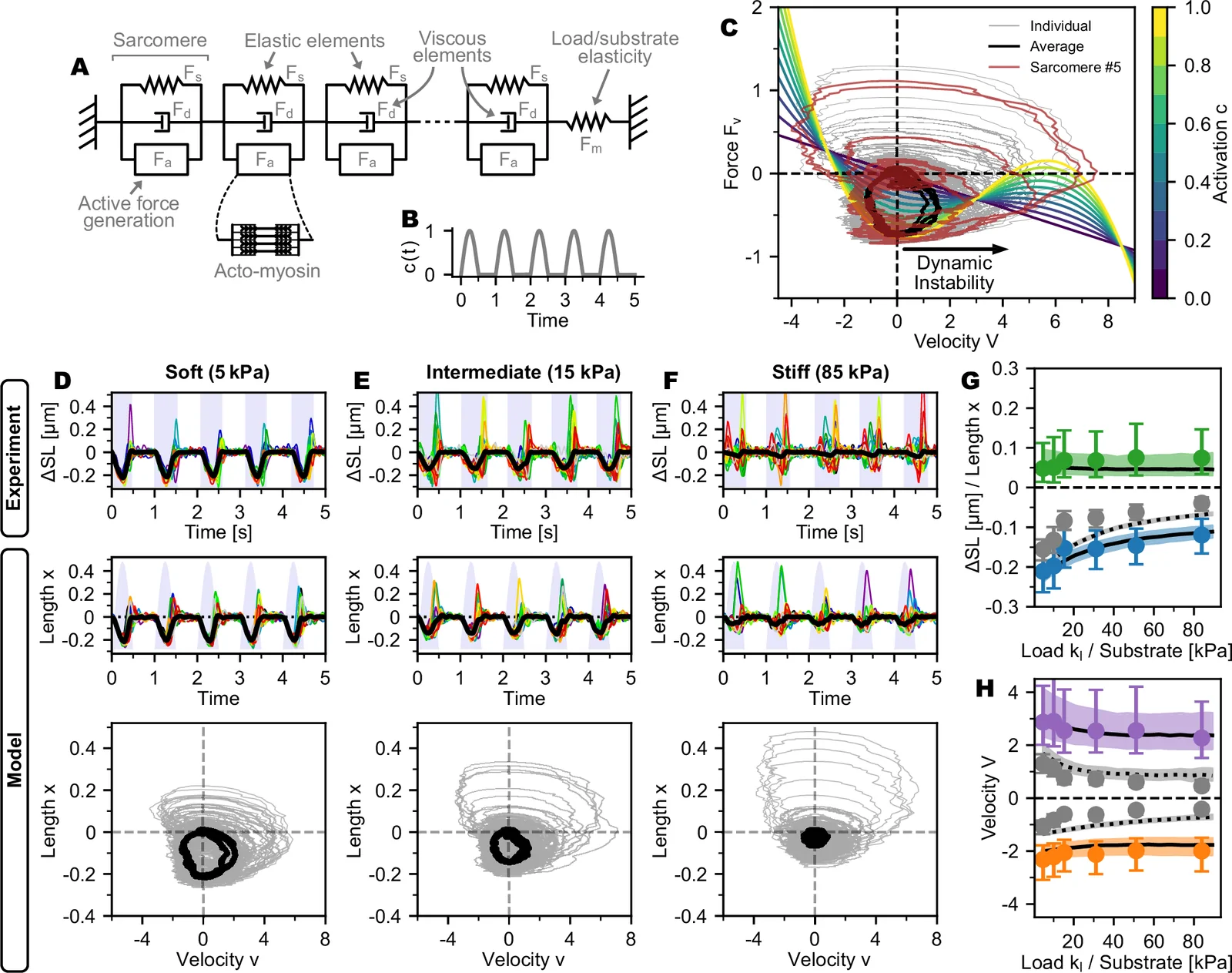
Mesoscopic model of coupled sarcomeres with non-monotonic force–velocity dynamics and comparison with data. (**A**) Schematic of the myofibril model where each sarcomere comprises three parallel components: an active force generator, creating force $F_{a}$, a viscous element creating force $F_{d}$, and a passive elastic element, creating force $F_{s}$. Sarcomeres are mechanically coupled in series; such that an external force $F_{m}$ generated by elastic substrate deformation is transmitted uniformly through all sarcomeres in the chain. (**B**) Activation imposes a time-dependent modulation of the active force throughout the contraction cycles, with a waveform $c\left (t\right)$. (**C**) Smooth curves: Activation-dependent force–velocity curves showing an S-shaped non-monotonic relationship with two stable branches and an unstable region with a negative-slope; the force–velocity relations are color-coded by activation level. Superimposed trajectories (gray) are trajectories of 20 individual sarcomeres, generated by Equation 2. The black trajectory is the myofibril average, and one sarcomere is highlighted in red. (**D–F**) Experimental recordings and corresponding model outputs for cells on soft (5 kPa), intermediate (15 kPa), and stiff (85 kPa) substrates showing timeseries of ΔSL/length *x* with blue-shaded contraction intervals (experiment) or $c\left (t\right)$ (model), together with the associated *v*–*x* trajectories in phase space; colored traces depict individual sarcomeres, and black traces denote cycle averages. (**G, H**) Extrema (maximum and minimum) of length *x* and velocity *v* per contraction cycle for individual sarcomeres and for the myofibril average as a function of substrate stiffness $k_{l}$; dashed lines indicate the average, solid lines show individual sarcomere values, colored shaded regions denote the standard deviation, and points with error bars display the experimental data median and inter-quantile range.

To rationalize the observed complex sarcomere dynamics, we developed a model (Figure 4A) describing the dynamics of each sarcomere i by an underdamped Langevin equation—a second-order stochastic differential equation. We represent a sarcomere mechanically as composed of three parallel components: an active force generator, contributing force $F_{a,i}$, a viscous damper, contributing force $F_{d,i}$, and an elastic element, contributing force $F_{s,i}$. Since we neglect physical mass, forces at all nodes between sarcomeres are instantaneously balanced, meaning every sarcomere experiences the same external load $F_{m}$. However, this external load is not instantaneously balanced by the internal forces ($F_{a},F_{d},F_{s}$) within each sarcomere due to the transient nature of the contraction. The resulting force imbalance distributes differently among the three elements from sarcomere to sarcomere, driving asynchronous dynamics (Figure 4A). To capture this non-equilibrium behavior, we introduce the effective parameter , which acts as a quasi-inertial mass. The equation of motion reads:

(2) $$\displaystyle \overset{˙}{v_{i}}=\frac{1}{\mu }\left [F_{a}\left (v_{i},c\right)+F_{d}\left (v_{i}\right)+F_{s}\left (x_{i}\right)-F_{m}\left (\overset{\rightarrow }{x}\right)\right ]+\eta \left (t\right),\quad\overset{˙}{x}_{i}=v_{i},$$

where $v_{i}$ and $x_{i}$ are velocity and length of sarcomere i, $\vec{x}$ is a vector of all sarcomere lengths, $c\left (t\right)$ is the time-dependent activation, and $\eta \left (t\right)$ a Gaussian noise term. Multiplying Equation 2 by recovers the form of Newton’s second law ($F\mathrm{=}m\cdot a$), where $\dot{v}_i$ represents the inertial term. While not a physical mass, models the characteristic time scale of the system’s kinetic response, reflecting the delayed attachment and detachment rates of actomyosin cross-bridges. This parameter defines how fast the system is drawn to the nullcline determined by the force balance, effectively smoothing instantaneous force fluctuations, representing the system’s persistence against rapid force changes.

This phenomenological underdamped Langevin framework, previously successfully applied to non-linear systems in general (Strogatz, 2018) and to cell migration in particular (Brückner et al., 2019; Brückner et al., 2021), captures the non-linear dynamics and emergent collective behaviors observed in our sarcomere system without explicitly modeling the underlying molecular mechanisms. This makes it particularly well-suited for studying the emergence of complex, large-scale behavior from fundamental single-sarcomere interactions.

To model the active actomyosin force $F_{a}$, we chose the third-order polynomial ansatz, while the viscous drag $F_{d}$ is modeled as a linear friction term:

(3) $$\displaystyle F_{a}\left (v_{i},x_{i},c\right)=\left (f_{0}+f_{1}v+f_{2}v^{2}+f_{3}v^{3}\right)\cdot c\left (t\right),$$

(4) $$\displaystyle F_{d}\left (v_{i}\right)=-\gamma _{s}v_{i}.$$

The polynomial in Equation 3 is a generalist functional form capable of capturing a broad spectrum of force–velocity relationships (Brückner et al., 2021; Brückner et al., 2020). Multiplying by the time-dependent activation $c\left (t\right)$ makes sure that active force generation scales with calcium availability, reflecting troponin C binding and the resulting availability of myosin binding sites on actin (Spudich, 2001). At high activation (c≈1), $F_{a}$ drives contraction, while at low activation (c≈0), the viscous drag $F_{d}$ dominates, governing passive relaxation dynamics.

In our model, as in previous work (Loescher et al., 2023), we represent the passive elastic element inside each sarcomere i using a piecewise defined function, allowing for different behaviors for shortening and lengthening. We here assume that elastic resistance during shortening is linear (provided by, e.g., microtubules Prosser, 2023) and quadratic during lengthening (provided by, e.g., titin Loescher et al., 2023):

(5) $$\displaystyle {\it F}_{s,i}(x_i)= \begin{cases} -k_1 \cdot x_i & \text{for } x_i < 0 \text{ (shortening)}\\ -k_1 \cdot x_i-k_2 \cdot x_i^2 & \text{for } x_i \geq 0 \text{ (lengthening).} \end{cases}$$

Here, $k_{1}$ and $k_{2}$ are elastic constants. For simplicity and without loss of generality, we set the sarcomere equilibrium length to zero ($x_{eq}=0$). For serially coupled sarcomeres, the total elastic force on each sarcomere is $F_{p,i}\left (\vec{x}\right)=F_{s,i}\left (x_{i}\right)-F_{m}\left (\vec{x}\right)$ where $F_{m}\left (\vec{x}\right)$ is the external myofibril load. For our experiment of single hiPSC-derived CMs on micropatterned elastic soft gels, we assume an auxotonic load $F_{m}\left (\vec{x}\right)=-k_{l}\cdot x_{m}$ with myofibril length change $x_{m}=\sum _{j}x_{j}$ and elasticity $k_{l}$.

We model stochastic fluctuations on the sarcomere level through the random noise term $\eta \left (t\right)=\eta _{t}\left (t\right)+\eta _{a}\left (t\right)$. This term combines two distinct components: thermal noise $\eta _{t}\left (t\right)$ and active noise $\eta _{a}\left (t\right)$, originating from the stochastic dynamics of the collective action of the myosin motors in the sarcomere. As shown previously (Nadrowski et al., 2004), these fluctuations arising from the stochastic motor kinetics can be approximated as delta-correlated:

(6) $$\displaystyle \left \langle \eta _{a}\left (t\right)\eta _{a}\left (0\right)\right \rangle \simeq \eta _{a,0}nr\left (1-r\right)\delta \left (t\right),$$

with system-specific magnitude $\eta _{a,0}$, the fraction of active motors *n* and the duty ratio of motors *r*. These fluctuations originate from the discrete, stochastic switching of individual motors between bound and unbound states (Nadrowski et al., 2004). For the active fluctuations, we model *n* with a sigmoidal function with steepness *β* dependent on velocity *v* dependent on activation $c\left (t\right)$, assuming active shortening and passive elongation of sarcomeres:

(7) $$\displaystyle n\left (v,c\right)=\frac{1}{1+{\rm exp}\left (-v\beta \right)}\cdot c\left (t\right).$$

### Data-driven parameter optimization reproduces complex sarcomere dynamics

Our model comprises 10 parameters. To specify the parameters, we simulated 20 serially connected sarcomeres over 10 contraction cycles using the Euler-Maruyama scheme (Kloeden and Platen, 1992). The cyclic activation was modeled using a simplified custom activation function *c*(*t*) based on a clipped sine with a beating rate matching the experimental data (Figure 4B). Since analytical fitting of such non-linear, stochastic and time-dependent systems to experimental data is impossible, we employed Differential Evolution (Storn and Price, 1997), a robust gradient-free optimization method, to identify the physical parameters generating the experimentally observed dynamics. The objective was to minimize a loss function based on the Kolmogorov–Smirnov (KS) coefficient, which quantifies the similarity between the distributions of simulated and experimental data on a scale from 0 (identical) to 1 (no overlap).

The model evaluation compared the distributions of sarcomere length changes and velocities from simulations with representative experimental LOIs from substrates (5, 15, and 85 kPa, mapped to *k_l_* = 0.5, 1.5, and 8.5 in our 1D model; $k_{l}$ is unitless, so only the ratios between values are meaningful—rescaling $k_{l}$ leaves model output unchanged under correspondingly rescaled parameters) covering the full range of mechanical loads. To account for the transient activation dependency, we divided the trajectories into four distinct phases: quiescent, early activation, mid-activation, and late activation, and compared the distributions for each phase using the KS metric. To match the experimental temporal resolution, model data was subsampled to 16 ms frame intervals. For each parameter set during optimization, all three substrate conditions were simulated, and the KS coefficient was computed for each condition and phase. The final loss was then calculated as the mean loss over all three conditions and four contraction phases, yielding a single metric quantifying the overall model-experiment agreement. The 10 model parameters were optimized within prespecified bounds (Supplementary file 1B).

This optimization process yielded a model in excellent agreement with the experimental data (Figure 4D–F). The average KS coefficient across all conditions and phases was ~0.05, confirming a high degree of similarity between the simulated and measured distributions of individual sarcomere lengths. Ultimately, we inferred a single, unified model capable of replicating dynamics across all substrate elasticities by adjusting only the substrate stiffness parameter. The model correctly captures the extremal trends (minima and maxima) of decreasing sarcomere displacement and velocity with increasing substrate stiffness at both the individual and average sarcomere levels (Figure 4G, H). While the model predicts slightly stronger average contraction and velocity extrema than measured, the stiffness dependence of these extrema is consistent with experimental observations (gray data in Figure 4G, H). Note that the model correctly replicates the emergence of smooth and periodic contraction at the myofibril level from the highly stochastic and heterogeneous dynamics of individual sarcomeres (Figure 4D–F).

### Non-monotonic force–velocity dynamics drive tug-of-war and limit cycles in coupled sarcomeres

Our data-driven inference procedure reveals, without making this an a priori assumption, that the active force $F_{a}$ exhibits a non-monotonic force–velocity relationship (Figure 4C, Video 2), with branches separated by an unstable region of negative slope ($dF\mathrm{/}dv{< }0$), corresponding to negative friction. This relationship is dynamically modulated by the activation $c\left (t\right)$, creating a complex time-dependent force landscape that governs sarcomere behavior.

**Video 2.** Computational model simulations showing the effect of substrate stiffness on sarcomere coordination. Simulations of sarcomere dynamics under three substrate stiffness conditions (resembling 5, 15, and 85 kPa). Top: Individual sarcomere length changes over time (colored traces) and the ensemble average (black trace). The thin vertical line indicates time progress. Bottom: Phase portraits in velocity–force space showing individual sarcomere trajectories (red traces), the ensemble average trajectory (black trace), and instantaneous sarcomeres at each time point (colored dots). The blue curve represents the force–velocity relationship (nullcline).

The model is effectively analogous to a Van der Pol relaxation oscillator in force–velocity space (Strogatz, 2018). As total myofibril shortening proceeds, the external mechanical load from the substrate increases, slowing down contraction velocities of all sarcomeres. The non-monotonic force–velocity relationship implies that there is an intrinsic threshold force at which sarcomeres become dynamically unstable. Physically, this corresponds to a tipping point where the stochastic unbinding of a few myosin cross-bridges triggers an avalanche-like release of the remaining more strongly loaded cross-bridges, switching the sarcomere from contraction to rapid elongation.

This instability has different consequences in the different phases of the contraction cycle: During the initial contraction phase, joint sarcomere shortening drives the velocity toward the instability threshold. Through dynamic selection, stochastic fluctuations (modeled by *η*) then push random sarcomeres past the tipping point. Because activation remains high, this elongation is transient; as a particular sarcomere lengthens, load quickly redistributes to its passive elements and while other sarcomeres take up the slack, it ends up back on the contracting branch. This rapid switching generates the stable limit-cycle high-frequency oscillations observed in individual sarcomeres (Figure 2H–J).

During relaxation (declining activation), the instability threshold decreases (Figure 4C). On stiff substrates, where external load remains high, this lower threshold causes a larger fraction of sarcomeres to slip past the instability point. Unlike in the first regime, the declining active force allows for large, sustained ‘popping’ excursions (Figure 4D–F). We observed that only a subset of sarcomeres pop (~10%), which effectively buffers the load for the remaining sarcomeres, allowing them to relax smoothly without overextension.

**Figure 5.**
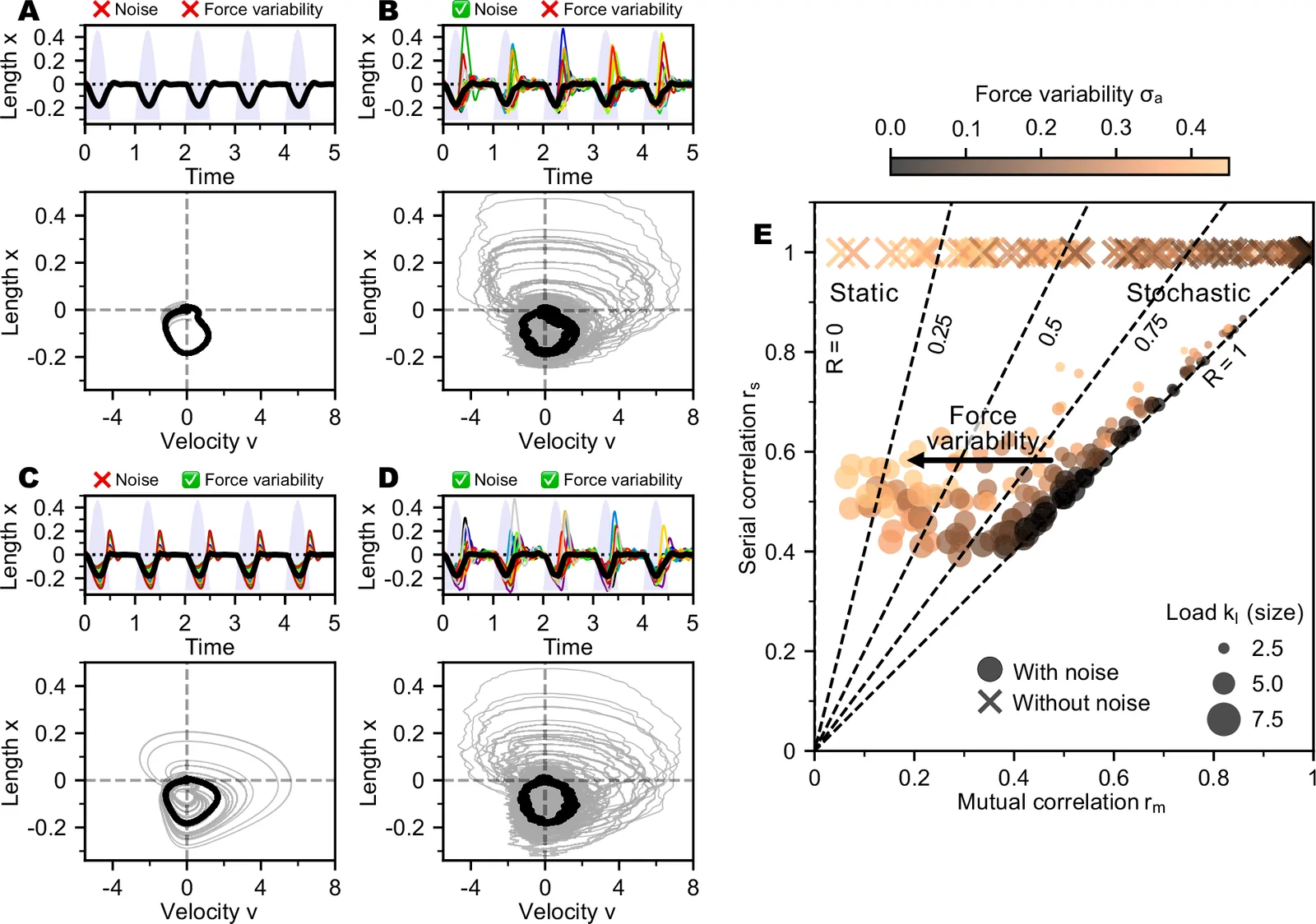
Simulation of static and stochastic heterogeneity in sarcomere dynamics. (**A–D**) Top panels: Overlaid timeseries of individual sarcomere length changes (colored curves) and the ensemble average (thick black curve). Bottom panels: Trajectories in phase space (length change vs. velocity) of individual sarcomeres (gray) and ensemble average (black). (**A**) Uniform ensemble (no noise, no force variability). (**B**) Stochastic heterogeneity driven by noise only. (**C**) Static heterogeneity driven by intrinsic force variability ($\sigma _{a}$) only. (**D**) Mixed, static and stochastic heterogeneity with both noise and force variability. (**E**) Serial correlation $r_{s}$ versus mutual correlation $r_{m}$ across load levels and force variability; marker size encodes the dimensionless simulated substrate stiffness $k_{l}$, and color shade indicates the standard deviation of the multiplicative factor modulating each sarcomere’s active force. Dashed lines mark the transition from purely static heterogeneity $\left (R\mathrm{=}0\right){\ }$ to purely stochastic heterogeneity $\left (R\mathrm{=}1\right)$. Circles denote simulations with noise/stochastic fluctuations, and crosses denote deterministic simulations without noise/stochastic fluctuations.

Stochastic fluctuations $\eta \left (t\right)$ (Equation 6) are essential to reproduce the heterogeneous sarcomere behavior seen experimentally, including beat-to-beat variability, competition, and popping events. Removing the stochastic drive from the equation of motion (Equation 2) in simulations collapses trajectories, inconsistent with experiments (Figure 5A, B). To probe robustness against fixed force non-uniformities, we introduced static variability by scaling each sarcomere’s active force $F_{a}$ with a constant factor drawn for each sarcomere in the chain of 20 from a distribution with standard deviation $\sigma _{a}$. Functionally, this assigns each sarcomere a slightly different maximum force threshold. This force variability alone biases trajectories and creates deterministic static heterogeneity during contraction according to each sarcomere’s strength (Figure 5C), also inconsistent with our data. Adding stochastic fluctuations leads to trajectories most closely resembling the experimental data (Figure 5D).

Correlation analysis (Figure 5E; compare with experimental data in Figure 3B) of the simulated data with different levels of static heterogeneity $\sigma _{a}$ shows, as expected, that without stochastic fluctuations, the serial correlation of sarcomeres is always 1, meaning that individual sarcomeres always behave the same way through contraction cycles. In contrast, simulations with stochastic fluctuations maintain stochastic heterogeneity (*R* > 0.8) even with substantial static force heterogeneity (up to $\sigma _{a}\approx 0.2$) (Figure 5E).

## Discussion

We combined high-speed imaging of human iPSC-derived CMs with detailed AI-based quantitative analysis. We discovered rich subcellular dynamics that remain hidden at the whole-cell level. While overall cell contraction cycles were regular, individual sarcomeres displayed rapid oscillations, reversible popping events, and stochastic beat-to-beat variability within the same myofibril. Increased substrate stiffness reduced overall cell contraction amplitudes but had only a weak effect on single-sarcomere length changes and contraction velocities. Rigid substrates instead promoted tug-of-war-like interactions and loss of synchrony between sarcomeres along myofibrils.

To interpret these observations mechanistically, we developed a data-driven, mesoscopic modeling framework describing transient, non-equilibrium dynamics and mechanical coupling among serially connected sarcomeres. The model relies on two components that accurately reproduce the experimentally observed dynamics: a non-monotonic force–velocity relationship that generates a dynamic instability at a critical force and intrinsic stochastic force fluctuations at the sarcomere level. This approach differs from classical modeling of skeletal muscle based on a force–length relationship. We find that our model is more appropriate than such models for CMs that operate in a regime of rapid transient activation–relaxation cycles, viscous drag, and velocity-dependent force generation.

The dynamic instability central to our framework arises from the non-monotonic shape of the force–velocity relationship. Note that this functional form was not an initial assumption but that it emerged when optimizing the parameters of the very general modeling ansatz to fit our experimental data. This result connects to foundational, reductionist theories of coupled molecular motors working against an external load (Jülicher and Prost, 1997; Guérin et al., 2010; Plaçais et al., 2009; Walcott and Sun, 2009), which predict that at a critical load, a collective, avalanche-like unbinding of motors from their track (e.g., myosin heads from actin in a sarcomere) induces a dynamic phase transition from active forward motion against the load to rapid passive slipping. Such instabilities have so far only been reported in reconstituted motility assays (Plaçais et al., 2009; Riveline et al., 1998). We have here observed an analogous instability in CMs manifesting itself in the observed switching between contraction and expansion and the popping events of individual sarcomeres. If sarcomeres followed a monotonic force–velocity relationship, increasing load would simply cause uniform slowing or stalling. Small non-uniformities caused by fluctuations would decay, returning the system to a uniform state. The non-monotonic force–velocity relationship, instead, amplifies such fluctuations, driving the system into limit cycles that produce the high-frequency oscillations and stochastic heterogeneity we observe (Figure 2G).

What could be the physiological roles of the observed phenomena? The popping events are the most dramatic outcome of the instability-amplified stochastic noise. Their reversible nature, ubiquity across all tested conditions, and prevalence at the end of contractions—where declining activation reduces the fraction of attached cross-bridges—are all consistent with a dynamic instability rather than structural failure. We therefore view popping as an intrinsic outcome of transient, non-equilibrium sarcomere dynamics. While possibly similar popping has been described in skeletal muscle (Morgan and Proske, 2006), particularly in the context of residual force enhancement (Johnston et al., 2016), the observation in rhythmically contracting CMs is a key new finding of this study. A physiological function of popping might be that intermittent yielding in a subset of sarcomeres accelerates myofibril-level relaxation toward the onset of the next diastole, helping tension decay even while individual sarcomere lengths remain non-uniform. A physiological role of stochastic heterogeneity of yielding might be the limitation of sarcomere damage. Our data indicate a mixture of static and stochastic heterogeneity. Some sarcomeres exhibit persistent differences suggestive of structural or compositional non-uniformities, yet many myofibrils show strong, cycle-to-cycle stochastic variability, including popping. As our simulations demonstrate, even in the presence of low to moderate sarcomere strength variability, the interplay between stochastic fluctuations and the dynamic instability prevents deterministic yielding. The interplay between intrinsic fluctuations and the dynamic instability randomizes yielding events across cycles and sarcomeres, preventing the repetitive overload of any single element and reducing the likelihood of localized damage.

Stochastic heterogeneity thus provides a robust mechanism for maintaining structural and functional homeostasis. Healthy adult CMs—with their more ordered myofibril and sarcomere architecture compared with stem-cell-derived CMs (Wadmore et al., 2021)—are expected to show smaller static non-uniformities while still exhibiting stochastic heterogeneity. While speculative for human CMs, this hypothesis is supported by in situ measurements of single-sarcomere dynamics in mouse myocardium, which found stochastic heterogeneity (Kobirumaki-Shimozawa et al., 2021).

In diseased and disordered myofibrils, static non-uniformities may overwhelm the protective stochastic mechanism (Månsson, 2014; Wadmore et al., 2021; Fomin et al., 2021) and instability may then be consistently triggered at the most vulnerable sarcomeres, concentrating mechanical stress and leading to fatigue and maladaptive remodeling. This perspective provides a more nuanced view than those based on fixed ‘weak–strong’ contrasts (Månsson, 2014), suggesting that pathology may arise from local structural weakness, but that that weakness would have to overwhelm the protective load-sharing mechanism. The phenomenon that amplified random noise can enhance responsiveness and stabilize dynamics has also been described in systems showing stochastic resonance (McDonnell and Abbott, 2009).

The presented framework is mesoscopic by design, omitting explicit molecular details such as calcium kinetics or individual cross-bridge states. Instead, we adopted a top-down approach, inferring the emergent mesoscopic physics directly from the experimental data. We utilized a mesoscopic Langevin framework in the hydrodynamic limit, where the deterministic force term represents the collective average of the underlying molecular kinetics and mechanical interactions, while the stochastic term accounts for unresolved microscopic fluctuations, following the general principles of active matter theory applied to actomyosin systems (Julicher et al., 2007; Prost et al., 2015). The strength of this coarse-grained approach is that it reveals emergent physical principles—such as the non-monotonic force–velocity relationship—that would remain obscured in parameter-heavy molecular models. The model’s predictive power, with only 10 parameters capturing dynamics across three substrate stiffnesses and four contraction phases, validates this strategy.

Our combined experimental and modeling platform offers a powerful tool to dissect the specific molecular drivers of collective sarcomere dynamics in future studies. By applying pharmacological perturbations or specific mutations, one could link distinct molecular defects—such as altered cross-bridge kinetics, calcium sensitivity, or titin stiffness—to quantitative changes in the model’s physical parameters. This would establish a rigorous mapping between molecular function and myofibril stability, enabling a systematic analysis of how specific pathologies disrupt the contractile homeostasis of the heart.

## Materials and methods

**Key resources table:** 

Reagent type (species) or resource	Designation	Source or reference	Identifiers	Additional information
Cell line (*Homo sapiens*)	TC-1133 (parental hiPSC line)	Baghbaderani, B. A. et al*.* cGMP-Manufactured Human Induced Pluripotent Stem Cells Are Available for Pre-clinical and Clinical Applications. *Stem Cell Reports* **5**, 647–59 (2015)	hPSCreg: RUCDRi002-A	https://hpscreg.eu/cell-line/RUCDRi002-A
Cell line (*Homo sapiens*)	ACTN2-Citrine reporter hiPSC, TC-1133-ACTN2-Citrine	Härtter et al., 2025	hPSCreg: RUCDRi002-A-3	Heterozygous C-terminal citrine knock-in at ACTN2
Peptide, recombinant protein	Synthemax II-SC substrate	Corning	Cat 3535	0.1 mg/ml, micropattern transfer
Chemical compound, drug	RPMI 1640 with GlutaMAX	Invitrogen	Cat 61870	Base of serum-free ‘cardio’ medium
Chemical compound, drug	B27 supplement	Invitrogen	Cat 17504-044	2% (vol/vol)
Chemical compound, drug	Penicillin/streptomycin	Invitrogen	Cat 15140	100 U/ml / 100 µg/m
Chemical compound, drug	StemPro Accutase cell dissociation reagent	Gibco	Cat A11105-01	Digestion medium
Chemical compound, drug	Trypsin	Gibco	Cat 15090-046	0.025% in digestion medium
Chemical compound, drug	DNase I	Calbiochem	Cat 260913	20 µg/ml in digestion medium
Chemical compound, drug	Y-27632 (Stemolecule, ROCK inhibitor)	Reprocell	Cat 04-0012-10	5 µmol/l
Chemical compound, drug	Acrylamide	Sigma-Aldrich	N/A	Gel solutions; elastic moduli per Supplementary file 1A
Chemical compound, drug	*N*,*N*′-Methylenebis(acrylamide)	Sigma-Aldrich	N/A	Crosslinker in gel solutions
Chemical compound, drug	Ammonium persulfate (APS)	Vizag Chemicals	N/A	Polymerization initiator, 1:100
Chemical compound, drug	Tetramethylethylenediamine (TEMED)	Muby Chemicals	N/A	Polymerization accelerator, 1:1000
Chemical compound, drug	Glutaraldehyde	N/A	N/A	0.5% (vol/vol) in ultrapure water; glass-slide coating
Software, algorithm	SarcAsM (Sarcomere Analysis Multitool)	Härtter et al., 2025	https://github.com/danihae/SarcAsM	Sarcomere detection and tracking; sarc-asm ≥0.4.0 (PyPI: sarc-asm)
Software, algorithm	SarcomereModel	This paper	https://github.com/danihae/SarcomereModel	Mesoscopic model of coupled sarcomeres; sarcomere-model v0.2.0, Python ≥3.10, <3.14
Other	6-Well culture plates	Corning	Cat 3516	Maintenance culture
Other	SU-8 photoresist, Series 3005	MicroChem	Series 3005	Photoresist masters for micropatterns
Other	PDMS (Sylgard 184 elastomer kit)	Dow Corning	Sylgard 184	10:1 base-to-curing-agent; stamps
Other	Silicon wafers	Microchemicals GmbH	N/A	Substrate for photoresist masters
Other	Custom photomasks	Compugraphics	N/A	Micropattern geometry
Other	Confocal microscope TCS SP5 II	Leica	N/A	8000 Hz resonant scanner, 67 fps
Other	Physica MCR 501 rheometer	Anton Paar	N/A	Measurement of gel elastic moduli
Other	Experimental dataset and analysis code	This paper	https://doi.org/10.5281/zenodo.17564384	Zenodo archive

### Cell lines

All experiments were performed with CMs derived from a single hiPSC line. The ACTN2-Citrine reporter line was generated as described previously (Härtter et al., 2025) from derivatives of the hiPSC line TC113345 (TC-1133; hPSCreg name RUCDRi002-A, https://hpscreg.eu/cell-line/RUCDRi002-A). The resulting line (RUCDRi002-A-3, TC-1133-ACTN2-Citrine; in short ACTN2-Citrine) was authenticated by genomic PCR and Sanger sequencing of the targeted ACTN2 locus, confirming correct in-frame integration of the citrine cassette in the parental genetic background. Cultures were tested routinely for mycoplasma by PCR and were consistently negative. Neither the parental nor the edited line appears on the Register of Misidentified Cell Lines maintained by the International Cell Line Authentication Committee (ICLAC).

### CM differentiation and culture

CM differentiation of the ACTN2-Citrine reporter hiPSC line (Härtter et al., 2025) was performed according to Tiburcy et al., 2017. hiPSC-ACTN2-Citrine-derived CMs were cultured in 6 well plates (Cat 3516, Corning) in serum-free ‘cardio’ medium 0.4 mM Ca^2+^, RPMI 1640 with GlutaMAX (Cat 61870, Invitrogen), 1% penicillin/streptomycin (Cat 15140, Invitrogen), 2% B27 supplement (Cat 17504-044, Invitrogen) at 37°C in a 5% CO_2_ incubator with culture medium changes at every other day. For re-seeding, cells were detached using Accutase digestion medium StemPro Accutase cell dissociation reagent (Cat A11105-01, Gibco), 0.025% Trypsin (Cat 15090-046, Gibco), 20 µg/ml DNase I (Cat 260913, Calbiochem) for 15–20 min at 37°C. Digestion was stopped using ‘cardio’ medium supplemented with 5 µmol/l Rock Inhibitor (Stemolecule Y27632, Cat 04-0012-10, Reprocell) three times the volume of the Accutase mix. Cell clumps were separated using a 100-µm cell strainer. Cells were seeded using ~150,000 cells per micropatterned substrate of ~1 cm^2^ size and cultured 24 hr in ‘cardio’ medium with 5 µmol/l Rock Inhibitor. CMs on soft gels were maintained in serum-free ‘cardio’ medium with daily medium changes at 37°C in a 5% CO_2_ incubator for up to 30 days. CMs were imaged after a maturation period of 20–30 days post seeding on the soft gels.

### Sample preparation and measurement

For live-cell video imaging, the soft substrates were mounted in a custom-built holder for round Ø25 mm glass cover slides with serum-free medium. Movies of beating CMs were obtained with a confocal microscope (TCS SP5 II, Leica, Germany) at 37°C and 5% CO_2_. We used an 8000 Hz resonant scanner with bidirectional scanning mode and recorded up to 20–30 s long movies of 1024 × 200 pixels at 67 frames per second, that is a temporal resolution of 15 ms.

### Fabrication of cell-adhesive micropatterns on polyacrylamide soft gels

Silicon wafers (Microchemicals GmbH, Ulm, Germany) were coated with SU-8 photoresist (Series 3005, MicroChem, Newton, USA) using a two-step spin-coating process. The wafers were then exposed to UV light through custom photomasks (Compugraphics, Jena, Germany) and developed to create photoresist masters (details in Härtter et al., 2025). Polydimethylsiloxane (PDMS) stamps were produced by mixing PDMS and curing agent 10:1 (Sylgard 184 kit, Dow Corning) and pouring it onto the photoresist masters. After degassing and curing, the PDMS was cut and peeled off to create the stamps. Micropatterned polyacrylamide gels were prepared by treating PDMS stamps with plasma to make them hydrophilic, then incubating them with 0.1 mg/ml Synthemax (Cat 3535, Corning). The protein-coated stamps were placed on plasma-cleaned glass coverslips and weighted to transfer the protein. Gel solutions of acrylamide and bis-acrylamide were prepared to achieve different elastic moduli (Supplementary file 1A), measured with a rheometer (Physica MCR 501 Rheometer, Anton Paar, Austria). Polymerization was initiated with ammonium persulfate (1:100, Vizag Chemicals) and TEMED (1:1000, Muby Chemicals), and the gel solution was polymerized on glutaraldehyde-coated glass slides (0.5% glutaraldehyde in ultrapure water) with the Synthemax-patterned glass on top. The gels were stored in PBS, then washed before use.

### Microscopy data analysis and statistical analysis

For processing of the live-cell confocal movies, and tracking and analysis of sarcomere trajectories, our Python package SarcAsM (Sarcomere Analysis Multitool) was used Härtter et al., 2025. All additional analyses and illustrations were created using custom scripts written in Python. All data is displayed as mean ± standard deviation unless indicated otherwise. Whenever applicable, we employed the Kruskal–Wallis test, a non-parametric method, for hypothesis testing, and proceeded to a post hoc multi-comparison using Dunn’s test, setting the threshold for statistical significance at a p-value of less than 0.05. All condition differences were deemed significant except where explicitly marked as not significant (n.s.).

### Computational model

We implemented the mesoscopic model of coupled sarcomeres in Python 3.12, making use of the NumPy (Harris et al., 2020) and SciPy (Virtanen et al., 2020) libraries. The model describes sarcomere dynamics through serially coupled underdamped Langevin equations, which is an approach suited to capturing emergent collective behaviors at the myofibril scale without explicitly modeling molecular-level details. We solved the system of stochastic differential equations numerically using the Euler–Maruyama scheme (Kloeden and Platen, 1992) with a time step of 2 ms. The dimensionless load parameter $k_{m}$ was provided as a simulation input, defined for simplicity as 0.1 times the substrate elasticity (in kPa). The remaining model parameters were estimated using Differential Evolution (Storn and Price, 1997), a global optimization algorithm, to minimize the Kolmogorov–Smirnov distance between simulated and experimental distributions.

## Data Availability

All data, analysis scripts, and source code supporting this study are publicly available. The primary software packages developed for this research, SarcAsM and the computational model, are available on GitHub at https://github.com/danihae/SarcAsM, copy archived at Härtter, 2026b and https://github.com/danihae/SarcomereModel, copy archived at Härtter, 2026a, respectively. The complete experimental dataset, alongside code to reproduce the analyses in this manuscript, is archived on Zenodo (https://doi.org/10.5281/zenodo.17564384). This repository includes a representative dataset of 25 raw microscopy movies with their corresponding processed line-of-interest (LOI) sarcomere trajectories for each of the six substrate stiffness conditions, as well as the aggregated data frame used to generate all figures. The following dataset was generated: HaertterD HaukeL 2025Supplementary data for: "Sarcomere dynamic instability and stochastic heterogeneity drive robust cardiomyocyte contraction"Zenodo10.5281/zenodo.17564384PMC1357433742734122

## Additional files

Supplementary file 1. Polyacrylamide gel compositions and model parameters. (A) Composition of polyacrylamide soft gels used in the study. All gels used here were made from the same respective stock solution (10 ml). The Young’s modulus was measured for a gel sample from each prepared stock solution using a rheometer (Physica MCR 501 Rheometer, Anton Paar, Germany) using a 25 mm, 2° cone plate with a sample volume of 140 μl. A time sweep (1 hr, spacing 30 s, 1% strain, 1 Hz), a frequency sweep (3 measurements per decade, 1% strain, 0.01–100 Hz), and an amplitude sweep (3 measurements per decade, 0.01–100% strain, 1 Hz) were performed consecutively. (B) Model parameters obtained through differential evolution optimization. Parameters were optimized by minimizing the Kolmogorov–Smirnov statistic between model simulations and experimental data from ACTN2-Citrine hiPSC-derived cardiomyocytes. Search ranges were based on physical constraints and preliminary analyses. Parameter values were rounded.

MDAR checklist
