# Supplementary material for: Sarcomere dynamic instability and stochastic heterogeneity drive robust cardiomyocyte contraction
Source: eLife. 2026 Sep 14;13:RP97321. doi: 10.7554/eLife.97321 (PMC13574337; doi:10.7554/eLife.97321)
Supplement: Supplementary file 1 [file elife-97321-supp1.pdf]

Supplementary file 1

Sarcomere dynamic instability and stochastic heterogeneity drive robust cardiomyocyte contraction

Daniel Haertter *et al.*

Table A. Composition of polyacrylamide soft gels used in the study. All gels used here were made from the same respective stock solution (10 ml). The Young's modulus was measured for a gel sample from each prepared stock solution using a rheometer (Physica MCR 501 Rheometer, Anton Paar, Germany) using a 25 mm, 2° cone plate with a sample volume of 140 µl. A time sweep (1 h, spacing 30 s, 1% strain, 1 Hz), a frequency sweep (3 measurements per decade, 1% strain, 0.01 – 100 Hz), and an amplitude sweep (3 measurements per decade, 0.01 – 100% strain, 1 Hz) were performed consecutively.

Young's modulus [kPa]	Acrylamide [ml]	Bis-acrylamide [ml]	PBS [ml]	Total [ml]
**5**	1.25	0.7	8.05	10
**9**	1.5	0.9	7.6	10
**15**	2.0	0.7	7.3	10
**29**	2.15	1.5	6.35	10
**49**	3.3	1.5	5.2	10
**85**	5.9	1.5	2.6	10

Table B. Model parameters obtained through differential evolution optimization.
Parameters were optimized by minimizing the Kolmogorov-Smirnov statistic between model simulations and experimental data from ACTN2-Citrine hiPSC-derived cardiomyocytes. Search ranges were based on physical constraints and preliminary analyses. Parameter values were rounded.

**Parameter**	**Symbol**	**Optimized Value**	**Search Range**	**Description**
Inertia	μ	6×10^{-3}	[0.0001, 0.01]	Pseudo-inertial parameter controlling adaptation timescale to force changes
Thermal noise	η_0	2.2	[0.5, 3.0]	Amplitude of thermal fluctuations (Gaussian noise)
Active noise	η_1	9	[1.0, 10.0]	Amplitude of active fluctuations from stochastic motor dynamics
Stiffness (linear)	k_{s,0}	2.5	[0.5, 5.0]	Linear elastic constant for sarcomere shortening and lengthening
Stiffness (quadratic)	k_{s,1}	3.2	[0, 10.0]	Quadratic elastic constant for sarcomere lengthening ($x > 0$)
Friction	u_{s,0}	0.10	[0.01, 0.2]	Passive friction within sarcomere (velocity-dependent)
F-V constant	f_{s,0}	-0.8	[-5, 5]	Constant term in polynomial force-velocity relation
F-V linear	f_{s,1}	-0.06	[-5, 5]	Linear coefficient in polynomial force-velocity relation
F-V quadratic	f_{s,2}	0.10	[-5, 5]	Quadratic coefficient in polynomial force-velocity relation
F-V cubic	f_{s,3}	0.012	[-5, 5]	Cubic coefficient in polynomial force-velocity relation
