# Supplementary material for: Sarcomere dynamic instability and stochastic heterogeneity drive robust cardiomyocyte contraction
Source: eLife. 2026 Sep 14;13:RP97321. doi: 10.7554/eLife.97321 (PMC13574337; doi:10.7554/eLife.97321)
Supplement: Peer review history

# Editor report: eLife Assessment

**DOI:** 10.7554/eLife.97321.4.sa0

**Authors:** Pascal Martin

**Affiliations:** Institut Curie France

**Keywords:** Compelling, Important

This **important** study provides a detailed characterization of individual sarcomeres' contractility and of their synchrony in spontaneously beating cardiomyocytes derived from human induced pluripotent stem cells. The combination of high-resolution tracking, statistical analysis and mesoscopic modeling leads to **compelling** evidence that sarcomeres operate as dynamically unstable units, leading to stochastic heterogeneities in their contraction-elongation cycles depending on substrate stiffness. The work will be relevant to scientists interested in muscle biophysics, nonlinear dynamics and synchronization phenomena in biological systems.

# Referee report: Reviewer #1 (Public review)

**DOI:** 10.7554/eLife.97321.4.sa1

[Editors' note: this version has been assessed by the Reviewing Editor without further input from the original reviewers. The authors have addressed the comments raised in the previous round of review.]

Summary:

In this manuscript, the authors present comprehensive experimental observations and a theoretical framework to explain the heterogeneous behaviour of sarcomeres in cardiomyocytes. They show that a stochastic component exists in their contractile activity, which may act as a feedback mechanism regulating physiological function.

Strengths:

Experiments and data analysis are robust and valid. The rigorous statistical analysis and unbiased methods enable the authors to draw well-supported conclusions that go beyond the existing literature. Their outcomes inform about cellular activity at the individual level and the authors explain how the transient dynamics of single sarcomeres are governed by a force-velocity relationship and lead to the complex contractile patterns. The similarity of the results to the study cited in [24] demonstrates the validity of the in vitro setup for answering these questions and the feasibility of such in-vitro systems to extend our knowledge of out-of-equilibrium dynamics in cardiac cells.

Very interesting the suggestion that the interplay between intrinsic fluctuations and the dynamic instability are part of a feedback mechanism for maintaining structural and functional homeostasis.

The addition of the theoretical model and the new text of the manuscript improves the clarity of the study.

# Referee report: Reviewer #2 (Public review)

**DOI:** 10.7554/eLife.97321.4.sa2

Summary:

Sarcomeres, the contractile units of skeletal and cardiac muscle, contract in a concerted fashion to power myofibril and thus muscle fiber contraction.

Muscle fiber contraction depends on the stiffness of the elastic substrate of the cell, yet it is not known how this dependence emerges from the collective dynamics of sarcomeres. Here, the authors analyze contraction time series of individual sarcomeres using live imaging of fluorescently labeled cardiomyocytes cultured on elastic substrates of different stiffness. They find that a reduced collective contractility of muscle fibers on unphysiologically stiff substrates is partially explained by a lack of synchronization in the contraction of individual sarcomeres.

This lack of synchronization is at least partially stochastic, consistent with the notion of a tug-of-war between sarcomeres on stiff sarcomeres. A particular irregularity of sarcomere contraction cycles is 'popping', the extension of sarcomers beyond their rest length. The statistics of 'popping' suggest that this is a purely random process.

Strengths:

This study thus marks an important shift of perspective from whole-cell analysis towards an understanding the collective dynamics of coupled stochastic sarcomeres.

# Referee report: Reviewer #3 (Public review)

**DOI:** 10.7554/eLife.97321.4.sa3

The manuscript of Haertter and coworkers studied the variation of the length of a single sarcomere and the response of microfibrils made by sarcomeres of cardiomyocytes on soft gel substrates of varying stiffness.

The measurements at the level of a single sarcomere are an important new result of this manuscript. They are done by combining the labeling of the sarcomeres z line using genetic manipulation and a sophisticated tracking program using machine learning. This single sarcomere analysis shows strong heterogeneities of the sarcomeres that can show fast oscillations not synchronized with the average behavior of the cell and what the authors call popping events which are large amplitude oscillations. Another important result is the fact that cardiomyocyte contractility decreases with the substrate stiffness, although the properties of single sarcomeres do not seem to depend on substrate stiffness.

The authors suggest that the cardiomyocyte cell behavior is dominated by sarcomere heterogeneity. They show that the heterogeneity between sarcomere is stochastic and that the contribution of static heterogeneity (such as composition differences between sarcomeres) is small.

Strengths:

All the results are, to my knowledge, new and original. The authors also made a theoretical model where each sarcomere is described by a Langevin equation based on a non-linear coupling between force and velocity of the sarcomeres. This model accounts well for the experimental results including the observation of what the authors call popping events.

# Author comment: Author response

**DOI:** 10.7554/eLife.97321.4.sa4

**Authors:** Daniel Haertter, Lara Hauke, Til Driehorst, Kengo Nishi, Wolfram H Zimmermann, Christoph F Schmidt

**Affiliations:** University Medical Center Göttingen Göttingen Germany; University Medical Center Göttingen Göttingen Germany; University of Göttingen Göttingen Germany; Duke University Durham United States; University Medical Center Göttingen Göttingen Germany; Duke University Durham United States

The following is the authors’ response to the previous reviews

**Public Reviews:**

**Reviewer #1 (Public review):**

Summary:

In this manuscript, the authors present comprehensive experimental observations and a theoretical framework to explain the heterogeneous behaviour of sarcomeres in cardiomyocytes. They show that a stochastic component exists in their contractile activity, which may act as a feedback mechanism regulating physiological function.

Strengths:

Experiments and data analysis are robust and valid. The rigorous statistical analysis and unbiased methods enable the authors to draw well-supported conclusions that go beyond the existing literature. Their outcomes inform about cellular activity at the individual level and the authors explain how the transient dynamics of single sarcomeres are governed by a force-velocity relationship and lead to the complex contractile patterns. The similarity of the results to the study cited in [24] demonstrates the validity of the in vitro setup for answering these questions and the feasibility of such in-vitro systems to extend our knowledge of out-of-equilibrium dynamics in cardiac cells.

Very interesting the suggestion that the interplay between intrinsic fluctuations and the dynamic instability are part of a feedback mechanism for maintaining structural and functional homeostasis.

The addition of the theoretical model and the new text of the manuscript improves the clarity of the study.

**Reviewer #2 (Public review):**

Summary:

Sarcomeres, the contractile units of skeletal and cardiac muscle, contract in a concerted fashion to power myofibril and thus muscle fiber contraction.

Muscle fiber contraction depends on the stiffness of the elastic substrate of the cell, yet it is not known how this dependence emerges from the collective dynamics of sarcomeres. Here, the authors analyze contraction time series of individual sarcomeres using live imaging of fluorescently labeled cardiomyocytes cultured on elastic substrates of different stiffness. They find that a reduced collective contractility of muscle fibers on unphysiologically stiff substrates is partially explained by a lack of synchronization in the contraction of individual sarcomeres.

This lack of synchronization is at least partially stochastic, consistent with the notion of a tug-of-war between sarcomeres on stiff sarcomeres. A particular irregularity of sarcomere contraction cycles is 'popping', the extension of sarcomers beyond their rest length. The statistics of 'popping' suggest that this is a purely random process.

Strengths:

This study thus marks an important shift of perspective from whole-cell analysis towards an understanding the collective dynamics of coupled, stochastic sarcomeres.

**Reviewer #3 (Public review):**

The manuscript of Haertter and coworkers studied the variation of the length of a single sarcomere and the response of microfibrils made by sarcomeres of cardiomyocytes on soft gel substrates of varying stiffness.

The measurements at the level of a single sarcomere are an important new result of this manuscript. They are done by combining the labeling of the sarcomeres z line using genetic manipulation and a sophisticated tracking program using machine learning. This single sarcomere analysis shows strong heterogeneities of the sarcomeres that can show fast oscillations not synchronized with the average behavior of the cell and what the authors call popping eveents which are large amplitude oscillations. Another important result is the fact that cardiomyocyte contractility decreases with the substrate stiffness, although the properties of single sarcomeres do not seem to depend on substrate stiffness.

The authors suggest that the cardiomyocyte cell behavior is dominated by sarcomere heterogeneity. They show that the heterogeneity between sarcomere is stochastic and that the contribution of static heterogeneity (such as composition differences between sarcomeres) is small.

Strengths:

All the results are, to my knowledge, new and original. The authors also made a theoretical model where each sarcomere is described by a Langevin equation based on a non-linear coupling between force and velocity of the sarcomeres. This model accounts well for the experimental results including the observation of what the authors call popping events.

We thank you and the reviewers for the positive evaluation of our revised manuscript.

**Recommendations for the authors:**

**Reviewer #2 (Recommendations for the authors):**

(1) Origin of the 3-Hz oscillation and required model extension. These oscillations are reproduced by our model, and their origin is already discussed in the manuscript (see lines 403–406).

(2) Inclusion of all 5085 LOIs vs. the selected 2321. We have expanded the explanation of the LOI selection criteria in the manuscript and clarified that the main conclusions are not sensitive to this choice (lines 161-166)

(3) Fig. 3G caption — popping rate. The caption has been updated to clarify the units and normalization.

(4) Fig. 4G — "Length x" vs. ΔL. Notation corrected for consistency.

(5) Fig. 4G — gray data points. Confirmed: these represent the mean, and the caption has been updated accordingly.

(6) Relation of k_l to the true substrate stiffness. We have added the following clarification: "The model evaluation compared the distributions of sarcomere length changes and velocities from simulations with representative experimental LOIs from substrates (5, 15, and 85 kPa, mapped to k_l = 0.5, 1.5 and 8.5 in our 1-D model; k_l is unitless, so only the ratios between values are meaningful — rescaling k_l leaves model output unchanged under correspondingly rescaled parameters) covering the full range of mechanical loads." (lines 365-369)

(7) Could a simpler model fit the data? The cubic polynomial in Eq. (3) was deliberately chosen as a generalist ansatz rather than imposed: its coefficients were obtained by data-driven inference via Differential Evolution, and if lower-order terms within this family had sufficed, the higher-order coefficients would have been driven toward zero. The inferred nonmonotonic force–velocity relation has two extrema separated by an unstable negative-slope branch, which sets a lower bound on the polynomial order — a linear F–v is monotonic and a quadratic admits only a single extremum, so cubic is the minimum polynomial order capable of producing the observed shape. Furthermore, the qualitative phenomena we report — popping events, dynamic instability, and stochastic heterogeneity — cannot arise from any monotonic force–velocity relation, as discussed in the section on the non-monotonic instability. With 10 parameters covering complex contractile dynamics at the individual sarcomere and myofibril level across different substrate stiffnesses, the present model is parsimonious within the family of polynomial force–velocity ansätze; we have not exhaustively searched alternative non-polynomial functional families, but any such alternative would still need to reproduce the same non-monotonic shape that the data require.

(8) Lines 497–507 in the Discussion. On reflection, we feel these lines provide useful context for the broader interpretation and would prefer to retain them.

(9) Line 331 — motivation of Eq. (3). We have added citations to prior work motivating this form of the equation for the broader readership.

(10) Line 427 — "scaled". Corrected.

**Reviewer #3 (Recommendations for the authors):**

We thank the reviewer for the recommendation of a theoretical appendix. The full model code, with the formulation and implementation documented in detail, is publicly available in our GitHub repository accompanying the paper, which we believe provides a complete reference for readers wishing to explore the model further. We therefore feel an additional appendix is not necessary within the scope of this revision.
